# Identifying Latent Profiles of Interpersonal Conflict Among Psychiatric Nurses and Exploring Their Associations With Psychological Capital, Sleep Quality, and Coping Styles: A Person‐Centered Study

**DOI:** 10.1155/jonm/2188621

**Published:** 2026-07-07

**Authors:** Ting Tang, Qiuxiang Sun, Qinghua Lu, Yuandong Gong

**Affiliations:** ^1^ School of Public Health, Shandong Second Medical University, Weifang, China; ^2^ Oncology Department, Weifang Hospital of Traditional Chinese Medicine, Weifang, China; ^3^ Department of Infection Management, Shandong Provincial Mental Health Center (Shandong University Mental Health Center), Jinan, China; ^4^ Shandong Provincial Key Medical and Health Laboratory of Digital Psychiatry, Shandong Provincial Mental Health Center (Shandong University Mental Health Center), Jinan, China

**Keywords:** interpersonal conflict, potential profile analysis, psychiatric nurse, psychological capital, sleep quality

## Abstract

**Aims:**

To explore the classification of interpersonal conflicts among psychiatric nurses and to compare the differences in psychological capital, sleep quality, and conflict coping styles among nurses with different conflict types.

**Methods:**

From July to August 2024, a survey was conducted among all clinical nurses in 6 tertiary psychiatric hospitals in Shandong Province using the General Information Questionnaire, Workplace Interpersonal Conflict Scale (WICS), Psychological Capital Questionnaire‐Revised (PCQ‐R), Simplified Coping Style Questionnaire (SCSQ), and Pittsburgh Sleep Quality Index (PSQI). Latent profile analysis was applied to explore the classification of interpersonal conflicts among psychiatric nurses. Univariate analysis and multivariate logistic regression analysis were used to identify the influencing factors of nurses’ conflict types.

**Results:**

The survey revealed that clinical psychiatric nurses could be divided into four types: low conflict‐adaptive type, moderate‐to‐high conflict‐clinical high‐pressure type, moderate‐to‐high conflict‐management contradiction type, and ultra‐high conflict‐omnidirectional high‐pressure type. Age, married status, total score of psychological capital, total score of sleep quality, and negative coping style of psychiatric nurses had significant effects on their conflict types.

**Conclusion:**

There is group heterogeneity in the conflict types of clinical psychiatric nurses. Poor sleep quality and negative coping styles increase the likelihood of belonging to higher‐risk conflict profiles, while psychological capital may not always function as a protective factor in interpersonal interactions. These findings highlight the importance of adopting person‐centered conflict management approaches for psychiatric nurses.

**Implication for Nursing Management:**

Nursing managers should pay more attention to nurses with moderate‐high conflict and ultra‐high conflict profiles. Measures such as improving the working environment, providing sufficient organizational support, and optimizing the scheduling system should be taken to reduce the incidence of interpersonal conflicts among psychiatric nurses. In addition, management should focus not only on enhancing psychological capital but also on fostering empathy and interpersonal collaboration skills to reduce conflict escalation and improve nursing care quality.

## 1. Introduction

Interpersonal conflict refers to a condition of tension, disharmony, hostility, and even confrontation among independent individuals [1]. Nursing is essentially a caring interpersonal interaction [2] and is characterized by high responsibility, high risk, and high intensity, making nurses a high‐risk group for interpersonal conflict. Studies have shown that interpersonal conflict is the most common type of conflict within nursing groups [3], and nurses are often central figures in various medical conflict situations.

Psychiatric nursing is an important branch of the nursing field. Its work environment has characteristics such as low patient cooperation, relatively enclosed ward settings, and a high incidence of violent incidents [4]. Additionally, the rate of workplace violence for nurses in mental health institutions is three times that of other nurses [5]. Therefore, psychiatric nurses need not only professional medical knowledge and skills but also the ability to meet patients’ emotional needs and prevent potential violence risks. Long‐term exposure to high‐risk environments makes psychiatric nurses prone to negative physiological, psychological, and behavioral changes, which aggravates job burnout and even creates strong turnover intention.

Psychological capital is a valuable resource for dealing with conflicts, composed of four psychological resources: self‐efficacy, hope, optimism, and resilience [6]. Self‐efficacy refers to a person’s belief in their ability to mobilize resources such as motivation, cognition, and action to meet various situational demands [7]. Studies have shown that nurses with high self‐efficacy have more optimistic mindsets and more positive energy and are more confident in handling interpersonal relationships and challenges within the team [8]. Hope is a prominent feature of high psychological capital, and nurses with high hope levels have more positive work attitudes, engagement, and concentration [9]. Optimism and resilience enable nurses to actively seek solutions when facing difficulties and challenges, rather than respond negatively or avoid them [10]. In addition to serving as an internal resource for individuals to cope with conflicts, psychological capital also promotes the construction, maintenance, and repair of positive relationships through a series of positive psychological and social processes, thereby creating a harmonious interpersonal environment. According to Fredrickson’s broaden‐and‐build theory of positive emotions, positive emotions can expand an individual’s cognitive and behavioral scope, enabling individuals to maintain more open and flexible states and hold relatively positive attitudes toward their surroundings [11]. Psychological capital continuously generates positive emotions [12], so nurses with high psychological capital are more willing to actively initiate social interactions, accept others’ viewpoints, and lay the foundation for good interpersonal relationships.

Coping style refers to the cognitive and behavioral strategies that individuals adopt when facing stress or difficulties, usually divided into positive coping and negative coping [13]. A positive coping style refers to the psychological and behavioral tendency for individuals to actively adopt positive behaviors to solve problems and reduce stress reactions when encountering stressful events or situations [14]. As a typical “support‐generating” behavior, positive coping style can increase the availability of social support and provide important support for building and strengthening interpersonal networks. A negative coping style refers to individuals avoiding difficulties or giving up on facing pressure through negative attitudes and behaviors, often manifested as self‐blame, complaints, withdrawal, and other emotional reactions [15]. Studies have confirmed that psychiatric nurses who tend to adopt positive coping styles have higher resilience levels, meaning these nurses can not only effectively cope with and handle stress and setbacks, but also effectively manage negative emotions such as frustration and anxiety in interpersonal interactions, thereby avoiding the transformation of personal stress into interpersonal hostility and maintaining good workplace interpersonal relationships.

Additionally, previous studies have shown that good sleep helps maintain stable neural circuits related to emotion regulation, promotes effective control of the amygdala by the prefrontal cortex, and enhances individuals’ emotion regulation ability [16]. Stable emotional states enable nurses to maintain rationality and empathy at work and understand patients’ behaviors and needs more deeply. These states are crucial for improving communication quality, reducing interpersonal conflicts, and creating harmonious nurse‐patient and team relationships. Studies have confirmed that there is a mutual influence between sleep quality and emotional states of nurses in psychiatric hospitals, that is, nurses’ anxiety or depression affects sleep quality, and in turn, poor sleep quality further worsens their anxiety or depression, thus forming a vicious cycle [17]. Work‐family conflict is also a factor affecting sleep quality [18], as tense family relationships and insufficient social support can affect sleep to some extent, while poor sleep in turn seriously affects nurses’ daily life and social activities. Therefore, good sleep quality is the foundation for nurses to actively carry out their work.

Previous studies on nurse conflicts mostly treated the nurse group as a homogeneous whole and failed to fully consider possible systematic differences between different subgroups within the group [19]. In reality, the interpersonal conflicts faced by different nurses vary in sources and forms of expression. For example, some nurses’ conflicts may mainly come from patients, while others may mainly come from internal team conflicts. This complex interpersonal conflict network cannot be ignored and requires a new research paradigm that can accurately capture individual differences for identification and analysis. Latent profile analysis (LPA), as a person‐centered statistical method, can identify potential subgroups with homogeneous characteristics based on individuals’ response patterns across multiple observed variables [20]. Based on this, this study uses LPA to identify potential conflict categories among psychiatric nurses and proposes the following hypotheses:

 H1: Psychological capital has a protective effect on interpersonal conflict among psychiatric nurses.
 H2: Poor sleep quality (high Pittsburgh Sleep Quality Index [PSQI] total score) is a risk factor for interpersonal conflict among psychiatric nurses.
 H3: Negative coping style is a risk factor for interpersonal conflict.

This study used the average scores of psychiatric nurses on the four dimensions of the Workplace Interpersonal Conflict Scale (WICS) to explore the latent profile characteristics of their interpersonal conflict. Then, through univariate analysis and multivariate logistic regression analysis, we identified key influencing factors associated with these unique conflict profiles, in order to provide a basis for psychiatric nursing managers to develop targeted conflict management strategies, thereby promoting the physical and mental health of psychiatric nurses, ensuring the quality and safety of mental health services, and ultimately achieving high‐quality nursing care.

## 2. Subjects and Methods

### 2.1. Participants

A multicenter convenience sampling method was used. From July to August 2024, one tertiary psychiatric hospital was selected from each of six geographic regions of Shandong Province (northwest, southwest, south, north, central, and peninsula areas). Clinical nurses from these hospitals served as the study participants.

Inclusion criteria: (1) having registered nurse qualifications at their institution; (2) nurses working in clinical wards; and (3) voluntary participants in the survey.

Exclusion criteria: (1) nonpermanent staff of the unit (such as advanced trainees and interns) and (2) nurses on leave for 3 months or more.

### 2.2. Ethical Considerations

This survey was approved by the Ethics Committee of Shandong Mental Health Center (Approval No. [37] of 2023). The questionnaire strictly followed the principle of confidentiality, and all participants provided informed consent and voluntarily took part in the study. During the review process, privacy was completely safeguarded. The study protocol followed the moral norms of the 1964 Declaration of Helsinki.

### 2.3. Methods

#### 2.3.1. General Demographic Data Questionnaire

This questionnaire was intended to retrieve basic information of all participants, including gender, age, years of work experience, marital status, education level, professional title, monthly income, conflict frequency, and shift work frequency (days per week).

#### 2.3.2. WICS

The WICS scale used in this study was originally cited from Fujiwara et al. [21] in their research on job burnout among Japanese home care workers. The scale was initially developed by Yatomi et al. [22] to assess interpersonal conflicts among staff in elderly care institutions, comprising three dimensions of conflict with supervisors, colleagues, and care recipients. Based on this, Fujiwara et al. developed a new dimension of “conflict with care recipients’ family members” by referring to the “conflict with care recipients” dimension, thus forming a scale with four dimensions and a total of 15 items.

To adapt this scale for psychiatric nurses in China’s healthcare context, this study followed Brislin’s classic cross‐cultural adaptation guidelines, using the scale items from Fujiwara’s English publication as the source material for translation. First, two native Chinese speakers fluent in English independently translated the English scale into Chinese. One translator had a medical background, and the other was a general translator without a medical background, to ensure the translated items were both accurate and easy to understand. Then, the research team reviewed and discussed the two Chinese versions, integrating them into a preliminary Chinese version through consensus. To verify whether the Chinese translation accurately reflected the original scale’s meaning, the research team invited another native English‐speaking bilingual expert who had not been exposed to the original English scale to back‐translate the preliminary Chinese version into English. Subsequently, an expert panel consisting of 2 nursing management experts and 3 senior psychiatric clinical nurses was formed. This panel reviewed each item’s semantic equivalence and cultural appropriateness by comparing the back‐translated version with the original English scale, thus forming the second Chinese version of the WICS. To test this Chinese scale’s language clarity, content relevance, and cultural appropriateness, the research team conducted a small‐scale pilot study. The team recruited 15 psychiatric nurses to independently complete the second Chinese version and provide written feedback on the clarity, comprehensibility, and relevance of the items. Based on the feedback, the research team optimized the wording of some items, finally forming the Chinese version used for formal data collection.

In this scale, participants rated each item using a 4‐point Likert scale. The total score was the sum of all item scores, with higher scores indicating higher levels of perceived interpersonal conflict among nurses. In this study, the overall Cronbach’s *α* coefficient for the scale was 0.889. The Cronbach’s *α* coefficients for the supervisor, colleague, patient, and patient’s family dimensions were 0.864, 0.740, 0.828, and 0.852, respectively.

#### 2.3.3. Psychological Capital Questionnaire‐Revised (PCQ‐R)

This scale was developed by Luthans et al. [6] in 2007. The Chinese translated edition was published in 2008 [23]. The scale includes four dimensions: self‐efficacy, hope, resilience, and optimism. Each dimension contains 6 items, with a total of 24 items. It uses a 6‐point Likert scale for evaluation, ranging from 1 (strongly disagree) to 6 (strongly agree). The scale has been widely used and shown good reliability and validity [6]. In this study, the Cronbach’s *α* coefficient was 0.933, and the Cronbach’s *α* coefficients for the four dimensions were 0.873, 0.888, 0.754, and 0.689, respectively.

#### 2.3.4. Simplified Coping Style Questionnaire (SCSQ)

This scale was developed by Xie [24] and contains a total of 20 items. The positive coping dimension includes 12 items, with a Cronbach’s *α* coefficient of 0.890. The negative coping dimension includes 8 items, with a Cronbach’s *α* coefficient of 0.780. The scale uses a 4‐point Likert scale for scoring, with higher scores indicating a higher tendency. In this study, the Cronbach’s *α* coefficients for the positive coping and negative coping dimensions were 0.862 and 0.809, respectively, and the overall reliability was 0.836.

#### 2.3.5. PSQI

This scale was developed by Buysse et al. [25] in 1989 and is widely used to assess subjects’ sleep quality in the past month. It includes 7 dimensions with a total of 19 items: subjective sleep quality, sleep latency, sleep duration, sleep efficiency, sleep disturbances, use of sleep medication, and daytime dysfunction. Each dimension uses a 4‐level scoring system from 0 to 3 points. The sum of all item scores is the PSQI total score, with a range of 0–21. A PSQI total score greater than 7 indicates sleep quality problems, and higher scores mean poorer sleep quality. The Cronbach’s *α* coefficient of this scale was 0.809 [26]. In this study, the Cronbach’s *α* coefficient of this scale was 0.761.

#### 2.3.6. Survey Method

Each participating institution designated dedicated survey staff who received unified training from the researchers with detailed explanations of the scale content and completion guidelines. Uniform instructions were used during data collection, and questionnaires were collected within 1 week after distribution. A total of 904 questionnaires were distributed, and 850 were returned. After excluding invalid questionnaires with a missing rate of over 5% for any variable, random responses, and duplicate submissions, 812 valid questionnaires were retained, with an effective response rate of 89.9%. Missing values in the remaining valid samples were imputed using the mean imputation method to ensure data completeness and facilitate subsequent statistical analysis.

### 2.4. Statistical Methods

Mplus 7.4 software was used to conduct LPA on nurses’ interpersonal conflict levels. The best model was selected based on model fit and difference testing. Model fit was assessed using three indices: Akaike Information Criterion (AIC), Bayesian Information Criterion (BIC), and sample‐size‐adjusted BIC (aBIC). Smaller values of these indices indicate better model fit. Differences between models were compared using the Lo–Mendell–Rubin adjusted likelihood ratio test (LMRT) and bootstrap likelihood ratio test (BLRT). When *p* < 0.05, it indicated that the k‐class model had better fit than the (*k* − 1)‐class model. Classification accuracy was evaluated using entropy, with values closer to 1 indicating more precise classification [27]. Subsequently, statistical analysis of the data was performed using SPSS 22.0 software. Normally distributed continuous data were expressed as mean and standard deviation (*x̄* ± *s*), non‐normally distributed continuous data were expressed as median and interquartile range, and categorical data were expressed as frequency and percentage. Differences among latent profile groups in categorical variables were compared using the chi‐square test, while differences in continuous variables were compared using analysis of variance. The effects of various factors on different latent profiles were evaluated using multivariate logistic regression analysis. The significance level was set at *α* = 0.05.

## 3. Results

### 3.1. General Demographic Characteristics of Participants

This study included a total of 812 psychiatric nurses. The mean age was (32.70 ± 8.07) years. The median years of work experience was 6.00 (3.00–15.00) years. Among them, 604 were female (74.38%) and 208 were male (25.62%). 77.59% of the nurses were married. The most common professional title was senior nurse, accounting for 36.20% of the total. The majority had a monthly income of 3000–5000 yuan, accounting for 47.29% of the total. 44.70% of nurses had never experienced conflicts, and 42.12% had experienced conflicts 1–5 times (Table 1).

**TABLE 1:** General demographic characteristics table (*n* = 812, Shandong, China).

Variables		*n*	Constituent ratio (%)
Gender	Male	208	25.62
	Female	604	74.38

Education level	Junior college degree or below	341	42.00
	Bachelor’s degree or above	471	58.00

Marital status	Married	630	77.59
	Single	182	22.41

Professional title	Nurse	283	34.85
	Junior nurse	294	36.20
	Senior nurse	193	23.78
	Deputy chief nurse or above	42	5.17

Monthly income	< 3000 yuan	243	29.93
	3000–5000 yuan	384	47.29
	> 5000 yuan	185	22.78

Conflict frequency	0 times	363	44.70
	1–5 times	342	42.12
	6–10 times	53	6.53
	≥ 11 times	54	6.65

### 3.2. LPA of Nurses’ Interpersonal Conflict Scores

#### 3.2.1. Results of LPA

This study sequentially fitted 1‐ to 5‐class latent profile models. The results showed that AIC, BIC, and aBIC values gradually decreased as the number of profiles increased. When the number of profiles was 4, entropy reached the maximum value (0.923), indicating the highest classification accuracy, and both LMRT and BLRT showed *p* < 0.05, indicating that the 4‐class model was significantly better than the 3‐class model with reliable model fit.

When testing the 5‐class model, although the difference was statistically significant (*p* < 0.05), the entropy value decreased from 0.923 (*k* = 4) to 0.890 (*k* = 5), suggesting a decline in classification quality. Additionally, the 5‐class model contained small classes with probabilities of 11% and 12%, which were difficult to interpret reasonably in the context of psychiatric nurses’ interpersonal conflict characteristics.

In contrast, the 4‐class model had more balanced class probabilities (24%/34%/25%/17%), and each class demonstrated clear and interpretable patterns of interpersonal conflict, which are meaningful for clinical practice and future intervention development. Therefore, after comprehensive comparison across all the above criteria, Model 4 was selected as the optimal classification result (Table 2).

**TABLE 2:** Model fit indices for the LPA of interpersonal conflict among psychiatric nurses.

Type	AIC	BIC	aBIC	Entropy	LMRT	BLRT	Class probability (%)
1	29554.390	29695.375	29600.107				
2	27049.775	27265.952	27119.875	0.899	< 0.001	< 0.001	67/33
3	26012.502	26303.871	26106.984	0.862	0.0004	< 0.001	33/42/25
4	25411.504	25778.065	25530.369	0.923	0.0043	< 0.001	24/34/25/17
5	24978.521	25420.274	25121.769	0.890	0.0312	< 0.001	36/11/20/22/12

#### 3.2.2. Category Naming

**FIGURE 1:**
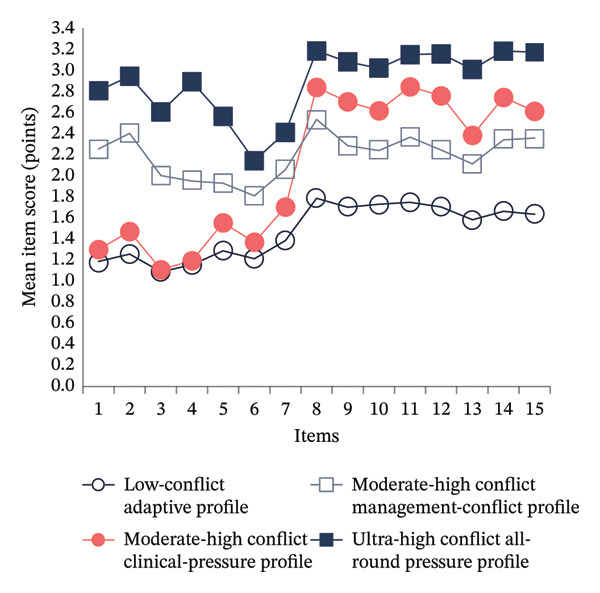
Four latent profiles of nurse conflict.

Based on the LPA results, nurses’ conflict patterns were divided into four classes. By calculating the mean scores of nurses in each class across the four dimensions of supervisor, colleague, patient, and patient’s family, we could analyze internal differences and thus name them appropriately. In Class 1, nurses had relatively low mean scores across all dimensions compared with other classes, so it was named the “Low Conflict‐Adapted Type.” In Class 2, nurses’ mean scores on the patient and patient’s family dimensions were significantly higher than other dimensions, while conflict scores with supervisors and colleagues were at relatively low‐to‐medium levels, so it was named the “Medium–High Conflict Clinical Pressure Type.” In Class 3, nurses’ mean conflict scores on the supervisor dimension were significantly higher than the first two classes, which was its most prominent feature. Although conflict scores with patients and their families were also at medium to high levels, they were not dominant. Therefore, this class was named the “Medium–High Conflict Management Conflict Type.” In Class 4, nurses’ mean scores across all dimensions were at the highest level among all classes, indicating they faced comprehensive conflict difficulties, so it was named “Ultra‐High Conflict All‐Round Pressure Type.” (Table 3) (Figure 1).

**TABLE 3:** Scores on multidimensional stressors and labels for the identified latent profiles.

Type	Mean score for each dimension of the questionnaire				Naming
	Supervisors	Colleagues	Patients	Patients’ families	
1	4.70	3.90	6.92	6.60	Low‐conflict adaptive profile
2	5.05	4.63	11.05	10.51	Moderate–high conflict clinical‐pressure profile
3	8.66	5.82	9.38	9.03	Moderate–high conflict management‐conflict profile
4	11.25	7.10	12.50	12.53	Ultra‐high conflict all‐round pressure profile

### 3.3. Univariate Analysis

There were statistically significant differences among nurses with different conflict types in marital status, age, years of work experience, total psychological capital score, total sleep score, positive coping score and negative coping score (*p* < 0.05) (Table 4).

**TABLE 4:** Univariate analysis across latent profile groups of nurse conflict (*n* = 812, Shandong, China).

Project		Low‐conflict adaptive profile (*n* = 198)	Moderate–high conflict clinical‐pressure profile (*n* = 280)	Moderate–high conflict management‐conflict profile (*n* = 199)	Ultra‐high conflict all‐round pressure profile (*n* = 135)	*χ* ^2^/*F*	*p*
Gender *n* (%)	Male	44 (22.22)	67 (23.93)	61 (30.65)	36 (26.67)	4.344	0.227
	Female	154 (77.78)	213 (76.07)	138 (69.35)	99 (73.33)		

Marital status *n* (%)	Married	144 (72.73)	225 (80.36)	163 (81.91)	98 (72.59)	7.999	0.046
	Single	54 (27.27)	55 (19.64)	36 (18.09)	37 (27.41)		

Education level *n* (%)	Junior college degree or below	93 (46.97)	114 (40.71)	78 (39.20)	56 (41.48)	2.855	0.415
	Bachelor’s degree or above	105 (53.03)	166 (59.29)	121 (60.80)	79 (58.52)		

Professional title *n* (%)	Nurse	87 (43.94)	93 (33.21)	60 (30.15)	43 (31.85)	15.823	0.071
	Junior nurse	69 (34.85)	106 (37.86)	74 (37.19)	45 (33.33)		
	Senior nurse	36 (18.18)	67 (23.93)	50 (25.13)	40 (29.63)		
	Deputy chief nurse or above	6 (3.03)	14 (5.00)	15 (7.53)	7 (5.19)		

Conflict frequency *n* (%)	0 times	100 (50.51)	116 (41.43)	90 (45.23)	57 (42.22)	10.421	0.317
	1–5 times	80 (40.40)	128 (45.71)	79 (39.70)	55 (40.74)		
	6–10 times	8 (4.04)	20 (7.14)	16 (8.04)	9 (6.67)		
	≥ 11 times	10 (5.05)	16 (5.72)	14 (7.03)	14 (10.37)		

Monthly income (yuan)	≤ 3000 yuan	73 (36.87)	76 (27.14)	53 (26.63)	41 (30.37)	10.315	0.112
	3000–5000 yuan	92 (46.46)	129 (46.07)	99 (49.75)	64 (47.41)		
	> 5000 yuan	33 (16.67)	75 (26.79)	47 (23.62)	30 (22.22)		

Age (*x* ± *s*)		30.46 ± 6.83	32.98 ± 7.93	34.11 ± 8.76	33.30 ± 8.40	7.611	< 0.001

Years of work experience *M* (IQR)		5.00 (2.75–10.00)	7.00 (4.00–13.75)	8.00 (4.00–20.00)	6.00 (3.00–19.00)	6.487	< 0.001

Shift frequency *M* (IQR)		2.00 (2.00–3.00)	2.00 (1.00–3.00)	2.00 (2.00–3.00)	2.00 (2.00–3.00)	0.746	0.525

Total psychological capital score (*x* ± *s*)		96.40 ± 20.20	102.35 ± 13.15	94.35 ± 16.91	97.15 ± 16.92	10.302	< 0.001

Total sleep score (*x* ± *s*)		5.42 ± 3.24	6.15 ± 3.62	6.71 ± 3.64	7.86 ± 3.81	13.451	< 0.001

Positive coping score (*x* ± *s*)		22.92 ± 6.93	24.33 ± 5.75	22.42 ± 5.63	22.73 ± 6.32	4.602	0.003

Negative coping score (*x* ± *s*)		8.45 ± 5.27	9.36 ± 4.37	10.45 ± 4.32	11.61 ± 4.45	14.823	< 0.001

### 3.4. Collinearity Diagnosis

Collinearity diagnosis was performed on continuous variables, using Tolerance < 0.1 and Variance Inflation Factor (VIF) > 10 as criteria for serious collinearity. The test found that the Tolerance values for age and years of work experience variables (0.116 and 0.120) were close to 0.1, and VIF values (8.595 and 8.367) were close to 10. Since years of work experience and age increase theoretically together, this study considered there was collinearity between age and years of work experience. Considering that the “age” indicator could reflect more comprehensive and objective information, the “age” variable was retained and the “years of work experience” variable was deleted (Table 5).

**TABLE 5:** Collinearity diagnostics.

Model 1	Unstandardized coefficient		Standardized coefficient	*t*	*p*	Collinearity statistics	
	*B*	SE	*β*			Tolerance	VIF
Constant	0.904	0.384		2.353	0.019		
Age	0.024	0.012	0.191	1.954	0.051	0.116	8.595
Years of work experience	−0.006	0.011	−0.058	−0.606	0.544	0.120	8.367
Shift frequency	0.016	0.021	0.027	0.765	0.445	0.877	1.140
Total sleep score	0.048	0.010	0.170	4.841	< 0.001	0.902	1.109
Positive coping	−0.015	0.006	−0.090	−2.308	0.021	0.725	1.380
Negative coping	0.048	0.008	0.221	6.354	< 0.001	0.916	1.091
Total psychological capital score	0.002	0.002	0.041	1.067	0.286	0.746	1.340

*Note:* Dependent variable: conflict type.

### 3.5. Multivariate Logistic Regression

The four nurse conflict types obtained from LPA were used as the dependent variable, with the low conflict adapted type as the reference group. Variables that showed statistical significance in the univariate analysis (marital status, age, total psychological capital score, total sleep score, positive coping styles, and negative coping styles) were entered as independent variables in the multivariate logistic regression analysis. The coding of ordinal variables is shown in Table 6. The results showed that age, total psychological capital score, total sleep score, and negative coping were risk factors affecting nurse conflict types (Table 7).

**TABLE 6:** Coding of independent variables.

Factor	Coding
Gender	Male = 1, Female = 2
Marital status	Married = 1, Single = 2
Education level	Associate degree and below = 1, Bachelor’s degree and above = 2
Professional title	Nurse = 1, Junior nurse = 2, Supervising nurse = 3, Associate chief nurse and above = 4
Monthly income (CNY)	≤ 3000 yuan = 1, 3000–5000 yuan = 2, > 5000 yuan = 3

**TABLE 7:** Logistic regression of factors associated with the four latent profiles of nurse conflict.

	Variables	*β*	SE	Wald *χ* ^2^	*p*	OR	95% CI
Moderate–high conflict clinical‐pressure profile	Constant	−4.67	0.813	32.073	< 0.001		
	Age	0.041	0.013	9.116	0.003	1.041	1.014∼1.069
	Total psychological capital score	0.27	0.007	15.017	< 0.001	1.028	1.014∼1.042
	Total sleep score	0.093	0.030	9.321	0.002	1.097	1.034∼1.165
	Negative coping	0.046	0.022	4.329	0.037	1.047	1.003∼1.094

Moderate–high conflict management‐conflict profile	Constant	−3.078	0.809	14.462	< 0.001		
	Age	0.064	0.014	20.496	< 0.001	1.066	1.037∼1.096
	Total sleep score	0.091	0.032	8.190	0.004	1.096	1.029∼1.167
	Negative coping	0.100	0.024	16.938	< 0.001	1.105	1.054∼1.159
	Married	0.529	0.244	4.720	0.030	1.698	1.053∼2.737

Ultra‐high conflict all‐round pressure profile	Constant	−5.545	0.965	33.034	< 0.001		
	Age	0.050	0.016	9.715	0.002	1.051	1.019∼1.084
	Total psychological capital score	0.020	0.008	6.017	0.014	1.020	1.004∼1.036
	Total sleep score	0.179	0.035	26.453	< 0.001	1.196	1.117∼1.280
	Negative coping	0.151	0.027	31.317	< 0.001	1.163	1.103∼1.226

*Note:* Low‐conflict adaptive profile served as the reference group; marital status was referenced to “unmarried.”

## 4. Discussion

### 4.1. Group Differences in Interpersonal Conflict Among Psychiatric Nurses

This study identified four conflict types among clinical psychiatric nurses using LPA: low conflict adaptive type (24%), medium‐high conflict clinical pressure type (34%), medium‐high conflict management contradiction type (25%), and ultra‐high conflict all‐around pressure type (17%). Based on the LPA of this study, 76% of psychiatric nurses experienced interpersonal conflicts of varying degrees. This proportion was significantly higher than the self‐reported incidence of interpersonal conflicts among nurses in the present study, which was 55.3% (the sum of the proportions of nurses reporting 1–5 times, 6–10 times, and ≥ 11 times of conflicts in Table 1). This suggests that psychiatric nurses may tend to underestimate or report their conflict experiences conservatively, while the actual conflict pressure at work is more widespread and hidden.

The medium‐high conflict clinical pressure type is the most common conflict type (34%). The possible reasons are that nurses, as frontline clinical staff, have heavy workloads and frequent contact with different patients and their families. This easily makes them physically and mentally exhausted, thus creating conditions for conflict events to occur. In addition, as people’s health awareness increases, patients and their families have higher expectations for medical care quality. However, they lack sufficient understanding of nursing work, which to some extent increases the frequency of conflicts. Second, psychiatry departments have more conflict events than other departments due to characteristics such as low patient cooperation and high risk of violence. Therefore, for nurses of the clinical conflict type, doctor‐patient communication training can be conducted to improve psychiatric nurses’ communication skills with patients. Establishing good doctor‐patient relationships helps to reduce clinical conflicts [28].

Notably, the medium–high conflict management contradiction type (25%) has a core characteristic of significantly prominent conflict with management, where leadership factors are central to the conflict. Research shows that leadership type has a significant influence on nurse conflict, and inclusive leadership can regulate nurse conflict. When its level is high, the work atmosphere among nurses is also higher [29]. Therefore, scientific leadership style is an important factor to ensure nurses’ normal work. In contrast, nurses of the low–conflict adaptive type (24%) have the lowest conflict scores in all four dimensions, showing good conflict‐handling ability. As top performers in conflict handling, managers can summarize their conflict handling experience for promotion and learning, thus comprehensively improving nurses’ conflict handling ability. Additionally, 17% of nurses experience comprehensive pressure from all sides, belonging to the ultra‐high‐conflict all‐around pressure type. This group has the most urgent need for intervention and the highest turnover risk and urgently needs systematic support.

### 4.2. Analysis of Influencing Factors

Results of multiple logistic regression analysis revealed key influencing factors that distinguish different profiles. These results confirmed Hypotheses 2 (H2) and Hypotheses 3 (H3) in the introduction, namely, that negative coping style and poor sleep quality are independent risk factors for interpersonal conflict among psychiatric nurses. This study unexpectedly revealed a positive correlation between psychological capital and two types of conflict among nurses. In addition, the study found that age and married status were significant independent influencing factors.

#### 4.2.1. The Special Nature of Psychological Capital

Unexpectedly, this study found that psychological capital level was positively associated with the moderate‐high conflict clinical‐pressure profile and the ultra‐high conflict all‐around pressure profile, which did not support Hypothesis 1 (H1). This finding suggests a more complex relationship between psychological capital and nurses’ interpersonal conflict, which may not follow the typical “protective resource” pattern observed in many previous studies.

Self‐efficacy is a pivotal constituent dimension of psychological capital. Bandura and Locke [30] argued that moderate self‐efficacy slightly exceeding actual capabilities yields beneficial effects, but severe overestimation may lead to persistent erroneous behaviors. In other words, excessively high self‐efficacy is likely to trigger overconfidence and make nurses rigid in clinical judgment, which readily escalates ideological divergences into interpersonal conflicts. Furthermore, high resilience is not positively correlated with enhanced empathy [31]. Nurses lacking empathy tend to underestimate others’ difficulties and emotional demands and are unable to deliver timely empathic care and emotional support in team cooperation and clinical communication. In the long run, emotional estrangement emerges between such nurses and their colleagues as well as patients, which imperceptibly elevates the risk of interpersonal disputes.

Additionally, nurses with high levels of psychological capital are generally tasked with more complicated assignments and frequently engage in intensive interpersonal communications and intersecting responsibility scenarios, which objectively increases the probability of conflict occurrence. As Chen et al.’s [32] research points out, psychological capital is positively correlated with nurses’ work engagement. Psychological capital has become an important personal resource that can improve employee performance, so it may be affected by managers’ staffing strategies.

This finding reminds nursing managers that psychological capital has a positive protective effect on nursing practitioners, which can effectively buffer occupational stress. Nevertheless, from the perspective of interpersonal conflicts and workplace interactions, excessively high psychological capital may give rise to rigid self‐positioning, thereby aggravating workplace interpersonal tensions. Therefore, while attaching importance to nurses’ individual psychological capital, medical institutions should also emphasize the cultivation of their interpersonal collaboration capabilities. By launching specialized training on conflict communication and emotional empathy, they can help nursing teams achieve dual development of sound mental health and harmonious workplace interpersonal relations.

#### 4.2.2. Poor Sleep Quality is a Comprehensive Risk Factor

The study found that poor sleep quality is a risk factor for three conflict types: the medium–high conflict clinical pressure type, the medium–high conflict management contradiction type, and the ultra‐high conflict all‐around pressure type. This supports H2. After controlling for other confounding factors, for each 1‐point increase in the total PSQI score, the likelihood of subjects being classified into these three conflict types was 1.097 times, 1.096 times, and 1.196 times the original, respectively. The mechanism is that the prefrontal cortex has functions such as executive control, emotion regulation, impulse control, and complex decision making [33]. When sleep quality is poor, the prefrontal cortex’s control over the amygdala weakens, making irritability, anxiety, and other emotions more likely to occur. At work, this manifests as poor concentration, reduced judgment, and worsened communication skills, leading to conflicts with patients, families, and colleagues. Particularly noteworthy is that sleep quality has the greatest impact on the all‐around pressure type nurses (OR = 1.196). For each 1 point decrease in sleep quality, the likelihood of falling into a completely uncontrolled state increases by 19.6%. This suggests that poor sleep quality may be an important factor driving the generalization of conflict.

Therefore, implementing measures focused on protecting and improving nurses’ sleep should be the cornerstone of conflict prevention. For example, managers can reduce nurses’ shift pressure by establishing reasonable scheduling systems and giving nurses more flexible free time. They can also improve nurses’ sleep quality through cognitive behavioral therapy, sleep education, and other methods to reduce conflict occurrence [34].

#### 4.2.3. Negative Coping Style and Conflict Escalation

This study shows that negative coping style is an important risk factor for interpersonal conflict among psychiatric nurses, which supports H3. Negative coping is essentially an ineffective stress management strategy. In this study, this factor shows a clear dose‐response gradient, that is, from clinical pressure type (OR = 1.047) to management contradiction type (OR = 1.105) and then to all‐around pressure type (OR = 1.163), with OR values increasing significantly in sequence. This suggests that there may be a vicious pathway for negative coping style. That is, when nurses choose to cope with conflicts negatively, unresolved clinical contradictions will further transform into higher‐level management contradictions. When facing escalating complex situations, individuals under great pressure turn to more extreme handling methods, eventually leading to a complete breakdown of work relationships and forming all‐around pressure. Previous research points out that positive coping is positively correlated with work life quality, while negative coping is negatively correlated with work‐life quality. Positive coping methods help nurses deal with daily pressure and face and solve problems directly [35].

Therefore, a negative coping style is not only a risk factor causing conflict but also the engine that drives conflict escalation and transformation. It is suggested that nursing managers can improve nurses’ psychological capital through art therapy [36]. They can also provide measures such as stress management courses and creating a supportive team atmosphere. These measures help nurses identify negative coping styles and transform them into positive coping styles, thereby enhancing nurses’ conflict resolution ability and blocking the escalation path of conflict.

#### 4.2.4. Age and Conflict Risk

This study found that age is a common risk factor for all conflict types. The possible reasons are that as age increases, nurses often become the backbone of their departments. In addition to routine work, they also face career pressures such as professional title promotion and continuing education [37], and they also need to balance family and work relationships. The combined pressures from multiple roles significantly increase their risk of conflict with patients, families, and colleagues in clinical work. Research by Hu et al. [38] points out that job burnout in the service industry is often accompanied by emotional fatigue, and this fatigue is especially obvious among middle and senior management. Older nurses have longer working years, and long‐term emotional labor and occupational exhaustion are accompanied by depletion of emotional resources, while their patience with patients and families also gradually decreases, thus making interpersonal conflict more likely to occur.

This finding reminds managers that high seniority is not a protective factor against conflict but may instead be a high‐risk signal of job burnout. Therefore, strengthening emotional guidance for senior nurses, providing psychological support services, and optimizing scheduling systems to relieve their occupational fatigue are important measures to reduce conflict occurrence.

#### 4.2.5. Married Status and Conflict Risk

This study found that married status is an independent risk factor for management contradiction type conflict in psychiatric nurses. Married nurses must bear responsibilities for caring for children and supporting elderly parents while also completing high‐intensity work. This role conflict continuously consumes their limited psychological resources, increasing emotional exhaustion and work dissatisfaction. Previous studies have found that married nurses generally face serious work‐family conflict [39], suggesting that imbalance between family and career roles may be the core trigger for management‐level contradictions. When work arrangements and family responsibilities create major conflicts, married nurses may be more sensitive to the fairness and care of management policies and are more likely to develop dissatisfaction, thus creating contradictions with managers.

Therefore, hospital management should pay attention to the special needs of married nurse groups. By implementing more humane flexible scheduling systems [40] and providing family support services to relieve married nurses’ role pressure, potential management contradictions can be reduced, thereby improving team stability and cohesion.

### 4.3. Limitations of Study

This study has several limitations. First, the cross‐sectional design of this study precludes any definitive conclusions about causality. Second, reliance on self‐reported measures may lead to potential common method bias, which could affect the relationships observed in this study. Third, as all data were collected from psychiatric hospitals in Shandong Province, China, our findings are specific to this regional context and may not generalize to other provinces or healthcare systems without further validation. Fourthly, in the collinearity analysis, removing the work experience variable, which directly reflects work proficiency and clinical skill accumulation, may have overlooked its independent predictive effect on different types of conflicts. Additionally, the retained age variable still cannot completely eliminate residual multi‐collinearity issues. Therefore, future studies could adopt Lasso regression to mitigate the interference of multicollinearity and maximize the independent explanatory value of key variables such as work experience and age. Future studies could conduct longitudinal or multi‐center research with larger sample sizes and broader sampling ranges, combined with qualitative research methods to deeply explore the influencing factors and formation mechanisms of each conflict type, in order to develop a more targeted nurse conflict management system.

## 5. Conclusions

This study used LPA to classify psychiatric nurses’ conflict types into four categories: low‐conflict adapted type, medium–high conflict clinical pressure type, medium–high conflict management conflict type, and ultra‐high conflict all‐round pressure type. This approach partially overcame the limitation of previous studies that often treated nurse groups as homogeneous and ignored internal differences. The study found that age, psychological capital level, sleep quality, and negative coping were important factors influencing conflict types among psychiatric nurses. The results suggest that nursing management should pay attention to group differences in conflict types and focus on the support needs of clinical pressure type, management conflict type, and all‐round pressure type nurses regarding sleep quality, psychological capital, and coping strategies. Through comprehensive measures such as improving environment, optimizing scheduling systems, conducting leadership training, and psychological empowerment, the risk of conflict occurrence and transformation can be reduced, thereby improving nursing service quality.

## Author Contributions

All authors contributed to the design of this study. Ting Tang: conceptualization, methodology, formal analysis, visualization, writing–original draft, and writing–review and editing; Qiuxiang Sun: investigation, data curation, methodology, and visualization; Qinghua Lu: conceptualization, data curation, and writing–review and editing; Yuandong Gong: supervision, project administration, funding acquisition, and writing–review and editing.

## Funding

The authors declare that financial support was received for this work and/or its publication. This work was supported by the “Shandong Province Health Human Resource Management Research Project (grant no. 2025RLZY004)” and the “Shandong Medical Staff Science and Technology Innovation Project (grant no. SDYWZGKCJH2023084).”

## Disclosure

This study was reported in accordance with the Strengthening the Reporting of Observational Studies in Epidemiology (STROBE) guideline for cross‐sectional studies.

## Ethics Statement

This study was approved by the Ethics Committee of Shandong Mental Health Center (Approval No. [37] of 2023). During the review process, privacy was completely safeguarded. The study protocol followed the moral norms of the 1964 Declaration of Helsinki.

## Conflicts of Interest

The authors declare no conflicts of interest.

## Data Availability

The data that support the findings of this study are available from the corresponding author upon reasonable request.
